# Altered Level of Consciousness in a Tertiary Emergency Department: Etiologies, Mortality, and Outcomes

**DOI:** 10.3390/jcm15052037

**Published:** 2026-03-07

**Authors:** Keun Tae Kim, Yong Won Cho

**Affiliations:** Department of Neurology, Keimyung University School of Medicine, Daegu 42601, Republic of Korea; neurocho@gmail.com

**Keywords:** consciousness disorders, etiology, emergency department, mortality, Glasgow Coma Scale, risk stratification

## Abstract

**Background/Objectives**: Altered level of consciousness (ALC) is a common emergency department (ED) presentation with high mortality. We evaluated etiologies and early ED-course prognostic markers for mortality. **Methods**: We retrospectively identified adult ED visits with ALC (September 2023–August 2025) and classified etiologies using the ALC-10 framework. Patients transferred directly to other hospitals were excluded because post-transfer outcomes were unavailable; sensitivity analyses were performed. Overall mortality was ED death or in-hospital death, and ED mortality was death during the ED stay. Nested logistic models were prespecified: overall-mortality Model A included age, initial Glasgow Coma Scale (GCS), etiologic category, and ICU admission, and Model B added vasopressor use and mechanical ventilation within 1 h; ED-mortality Model A included age and initial GCS, and Model B added vasopressor use and mechanical ventilation. **Results**: ALC accounted for 2.85% (2194/76,957) of adult ED visits; 1932 patients were analyzed after excluding 262 transfer-outs. Systemic infection (25.8%) and metabolic causes (23.7%) were most frequent. Observed overall mortality was 23.6% (455/1932), including ED mortality of 6.4% (124/1932); model-based sensitivity analysis estimated adjusted overall mortality to be 23.2% (95% uncertainty interval, 22.9–23.7) among all ALC visits. In adjusted models, older age, lower initial GCS, and vasopressor use were associated with higher odds of both outcomes, while ICU admission and mechanical ventilation were associated with overall mortality. Model B showed improved discrimination (AUC 0.795 overall; 0.869 ED). **Conclusions**: These findings highlight the prognostic significance of age, initial neurologic status, and etiology. This study may assist in risk stratification and early resource allocation.

## 1. Introduction

Altered level of consciousness (ALC), often described clinically as altered mental status, is a common, high-acuity presentation in the emergency department (ED). It ranges from mild confusion to coma and often requires simultaneous stabilization, diagnostic evaluation, and disposition decisions under diagnostic uncertainty. Because ALC is a syndrome rather than a single diagnosis, timely identification of reversible or time-critical etiologies is essential to prevent irreversible neurologic injury and death [1,2].

The etiologic spectrum of ALC is heterogeneous and varies across populations, case definitions, and clinical settings. ED-based cohorts and systematic reviews of acute coma indicate that both primary neurologic catastrophes and extracranial critical illnesses contribute substantially, with marked variability in the relative frequency of stroke, infection, metabolic derangements, intoxication, trauma, and psychiatric disorders [3,4,5,6,7,8]. This heterogeneity complicates bedside triage and limits cross-study comparability, underscoring the need for structured, reproducible etiologic classification.

In recent studies, a standardized ten-category framework (ALC-10) has been proposed and applied using a structured, consensus-based approach, enabling systematic description of etiologies and outcomes in both single-center and multicenter ED cohorts [9,10]. Mortality remains a key outcome in ALC; however, death during ED care represents a distinct endpoint that may reflect both extreme illness severity and ED-based end-of-life care needs. Nationwide Korean data indicate that ED deaths are predominantly disease-related and commonly involve prolonged ED stays and frequent cardiopulmonary resuscitation [11]. Studies from other health systems similarly report that ED death is not rare [12,13]. Separating ED mortality from in-hospital mortality may therefore clarify distinct clinical trajectories and identify time-sensitive opportunities for intervention.

System-level pressures may further influence outcomes in ED patients with ALC. Post-coronavirus disease-2019 (COVID-19) ED crowding and boarding have been associated with higher short-term mortality [14,15,16,17], and overnight ED stays among older adults awaiting ward beds have been associated with higher in-hospital mortality [17,18]. Meanwhile, the COVID-19 pandemic altered ED utilization patterns, and altered mental status has been recognized as an important presentation among patients with COVID-19 [19,20,21].

Against this background, we conducted a contemporary single-center cohort study of adults presenting to a tertiary ED in South Korea with ALC, classified using the ALC-10 framework [10]. We aimed (1) to quantify the incidence of ALC among adult ED visits; (2) to describe the current etiologic distribution of ALC using ALC-10; and (3) to characterize ED, in-hospital, and overall mortality and to evaluate early ED-course prognostic markers associated with death across the spectrum of ALC. By explicitly separating ED mortality from in-hospital mortality and focusing on variables available early in ED care, this study aims to provide an updated epidemiologic description and clinically actionable risk stratification for ALC in a high-acuity tertiary ED.

## 2. Methods

### 2.1. Study Design and Setting

This single-center, retrospective observational cohort study was conducted in the ED of a tertiary university hospital in South Korea. The ED provides 24-h emergency care and functions as a regional referral center. The Glasgow Coma Scale (GCS) was assessed at ED arrival by either the chief resident or an attending emergency physician.

### 2.2. Study Population and Definition of Altered Level of Consciousness

We screened all adult patients (≥18 years) presenting to the ED with ALC between 1 September 2023 and 31 August 2025. ALC was defined as an acute impairment in level of consciousness, orientation, attention, or responsiveness documented at ED presentation. A visit was considered an ALC case if either of the following criteria was met: (1) an initial GCS score < 15 at ED presentation or (2) an explicitly documented acute change in mental status compared with the patient’s presumed usual (premorbid) mental status (e.g., new-onset significant disorientation, hallucinations, or bizarre/inappropriate behavior).

The exclusion criteria were: (1) age younger than 18, since the etiologies of ALC in children are different from those in adults; (2) any inter-hospital transfers during an ongoing hospitalization at an external hospital, because pre-existing ALC (e.g., dementia, major stroke, or psychiatric illness) could not be reliably assessed or excluded; (3) dead on arrival or cardiac arrest on arrival because the cause of ALC could not be ascertained; and (4) a revisit within 48 h to avoid within-patient clustering.

### 2.3. Data Collection

We abstracted clinical data from the electronic medical record using a predefined data extraction form. Collected variables included age, sex, initial GCS score, ED length of stay, ED disposition, and early ED-course management-intensity markers (i.e., orders for mechanical ventilation or vasopressors entered in the electronic medical record within 1 h of ED arrival). ED disposition was categorized as discharge home, admission to a general ward (GW), admission to an intensive care unit (ICU), transfer to another hospital, or death in the ED. Patients transferred directly from the ED to another hospital were excluded from outcome analyses because post-transfer outcomes were unavailable. For admitted patients, in-hospital outcomes were collected, including length of hospitalization (days) and in-hospital mortality. There were no missing data for the variables used in the primary and secondary analyses; therefore, complete-case analyses were performed without imputation.

### 2.4. Etiologic Classification of ALC

The final etiology of ALC was assigned using a 10-category classification system proposed in prior studies of ALC in emergency settings [9,10]. Each case was classified into one of the following categories: Systemic Infection, Metabolic Cause, Stroke, Cardiogenic and Vascular cause (C&V), Seizure, Toxic, Psychiatric Disorder, Central Nervous System Infection (CNS-i), Traumatic Brain Injury (TBI), or Undetermined.

Systemic Infection refers to any infectious condition (e.g., sepsis, septic shock, pneumonia, urinary tract infection, and intra-abdominal infection) except CNS-i. Metabolic Cause included neurological disorders not caused by primary cerebral structural lesions, such as hepatic encephalopathy, uremic encephalopathy, electrolyte imbalance, and other non-infectious metabolic derangements. Stroke encompassed ischemic as well as hemorrhagic stroke (e.g., subarachnoid hemorrhage, intracerebral hemorrhage, subdural hemorrhage, and epidural hemorrhage) and included transient ischemic attack and transient global amnesia. C&V included acute coronary syndromes, clinically significant arrhythmias, heart failure, aortic dissection, and cardiogenic shock (i.e., ALC attributable to disease of the heart or aorta). Seizure included ongoing seizure, post-ictal states, and status epilepticus; psychogenic non-epileptic seizure (clinically established or documented) was classified within this category. Toxic exposure encompassed alcohol intoxication, drug overdose, and exogenous poisoning. Psychiatric Disorder was diagnosed by a psychiatrist after a face-to-face evaluation and exclusion of alternative causes. CNS-i included meningitis, encephalitis, and meningoencephalitis; in this study, the prerequisite of CNS-i was CSF WBC > 5/mm^3^. TBI referred to head trauma with structural brain injury documented on neuroimaging and judged to be responsible for ALC, regardless of surgical intervention. Undetermined encompassed ALC cases in which no single predominant etiology could be identified, including truly unknown causes after comprehensive evaluation, or not classified elsewhere. For etiologic categorization, a multidisciplinary consortium consisting of board-certified university professors in emergency medicine, internal medicine, and neurology held regular meetings. Consortium members presented individual cases in rotation, reviewed available clinical information, and assigned the etiology by consensus. When initial opinions differed, disagreements were resolved through further discussion until consensus was achieved. The etiology of ALC was determined based on the information available at the time the patient departed the ED.

### 2.5. Outcomes

Mortality was evaluated at two time points. ED mortality was defined as death during the ED stay before admission to the hospital or transfer to another hospital. In-hospital mortality was defined as death after admission from the ED (to a GW or ICU) and before hospital discharge (home or interhospital transfer). Patients transferred directly to another hospital from the ED (before admission) were excluded from outcome analyses because post-transfer outcomes were unavailable. Nonetheless, we compared ED-presentation characteristics of transfer-out patients with those of the analytic cohort to quantify potential selection bias introduced by this exclusion. Overall mortality was defined as the combined number of ED deaths and in-hospital deaths divided by the number of patients included in outcome analyses.

### 2.6. Statistical Analysis

All statistical analyses were conducted in R version 4.5.2 (R Foundation for Statistical Computing, Vienna, Austria). Continuous variables were summarized as mean ± standard deviation or median (interquartile range), and categorical variables were summarized as counts and percentages. Group comparisons were conducted using the chi-square test or Fisher’s exact test for categorical variables and the independent *t*-test or Mann–Whitney U test for continuous variables, as appropriate. Mortality differences across the ten etiologic categories were assessed using the chi-square test. Etiology-specific mortality proportions (ED mortality, in-hospital mortality among admitted patients, and overall mortality) were reported with 95% confidence intervals (CIs) calculated using the Wilson score method. All statistical tests were two-tailed, and *p*-values < 0.05 were considered statistically significant. This observational cohort study is reported in accordance with the STROBE guidelines.

In this study, we refer to candidate variables collectively as early ED-course prognostic markers, encompassing information available at ED arrival and markers accrued during the initial ED course (including early management intensity and ED disposition). These markers are used for pragmatic risk stratification and are not intended to support causal inference regarding treatment or disposition effects. To evaluate multivariable-adjusted associations with overall mortality (ED death or in-hospital death) and to contrast prognostic information available at ED arrival with information accruing during the early ED course, we prespecified a nested modeling strategy using early ED-course prognostic markers. For overall mortality, Model A included age, initial Glasgow Coma Scale (GCS) score, etiologic category (modeled as a categorical variable with systemic infection as the reference group), and ICU admission. Model B (augmented) additionally incorporated early ED-course management-intensity markers—vasopressor use and mechanical ventilation (orders entered in the electronic medical record within 1 h of ED arrival). For ED mortality, Model A included ED-arrival markers (age and initial GCS score), and Model B (augmented) additionally included vasopressor use and mechanical ventilation within 1 h of ED arrival (orders entered in the electronic medical record). These management-intensity markers were included as pragmatic proxies of early physiologic derangement and care intensity and should not be interpreted as having direct causal effects on mortality. Adjusted odds ratios (ORs) and 95% confidence intervals (CIs) were calculated. Multicollinearity was assessed using variance inflation factors (VIFs). Model discrimination was assessed using the area under the receiver operating characteristic curve (AUC), and bootstrap 95% CIs were estimated for the AUC. Model calibration was assessed using the Hosmer–Lemeshow goodness-of-fit test and visualized using calibration plots based on deciles of predicted risk (Supplementary Figures S1 and S2). The global significance of etiologic category was evaluated using a likelihood-ratio test.

To address potential bias from unascertained outcomes after direct inter-hospital transfer from the ED (transfer-out), we performed best- and worst-case sensitivity analyses by assuming mortality among transfer-out patients was 0% and 100%, respectively. In addition, we conducted a model-based sensitivity analysis for transfer-out patients. We fit a multivariable logistic regression model for overall mortality among patients with observed outcomes and applied it to transfer-out patients to estimate the expected number of deaths. The model included age, sex, initial GCS score, etiologic category (categorical; systemic infection as the reference), and early ED-course management-intensity markers. Expected deaths among transfer-out patients were calculated as the sum of individual predicted probabilities ($\sum \hat{p}i$), and adjusted overall mortality among all ED visits for ALC was calculated as (observed deaths + $\sum \hat{p}i$)/N. Uncertainty intervals were obtained from 10,000 simulations by sampling regression coefficients from their asymptotic multivariate normal distribution and taking the 2.5th–97.5th percentiles of the simulated distribution (Supplementary Table S5).

Because post-transfer mortality was unobserved, ED transfer-out represents loss to follow-up for patient-level mortality rather than a fully ascertained competing terminal event. A competing-risks framework would instead estimate the cumulative incidence of death during care at our institution (i.e., before transfer-out), which corresponds to a different estimand than overall mortality after ED presentation.

Among admitted patients (GW + ICU), we performed time-to-event analyses for in-hospital mortality using days of hospitalization as the time scale; discharge alive and in-hospital transfer were treated as censoring events. We conducted additional prespecified analyses to address reviewer requests: (i) propensity-score (PS) weighting with inverse probability of treatment weighting targeting the average treatment effect on the treated (IPTW-ATT) to compare ICU versus GW while balancing covariates measured at ED presentation (age, sex, and initial GCS), with balance assessed by standardized mean differences (SMD) and extreme-weight trimming (to ensure adequate covariate overlap, IPTW-ATT analyses were restricted to the region of common support by excluding admitted patients with propensity scores outside the overlap between ICU and GW groups, yielding N = 1348 for IPTW-weighted analyses); (ii) sensitivity analyses examining alternative GCS parameterizations and handling of the undetermined etiology category; (iii) a GCS × etiology interaction analysis; and (iv) a competing-risks analysis using the Fine–Gray subdistribution hazards model treating discharge alive and in-hospital transfer as competing events. These analyses are reported in Supplementary Tables S6–S11.

## 3. Results

**Figure 1:**
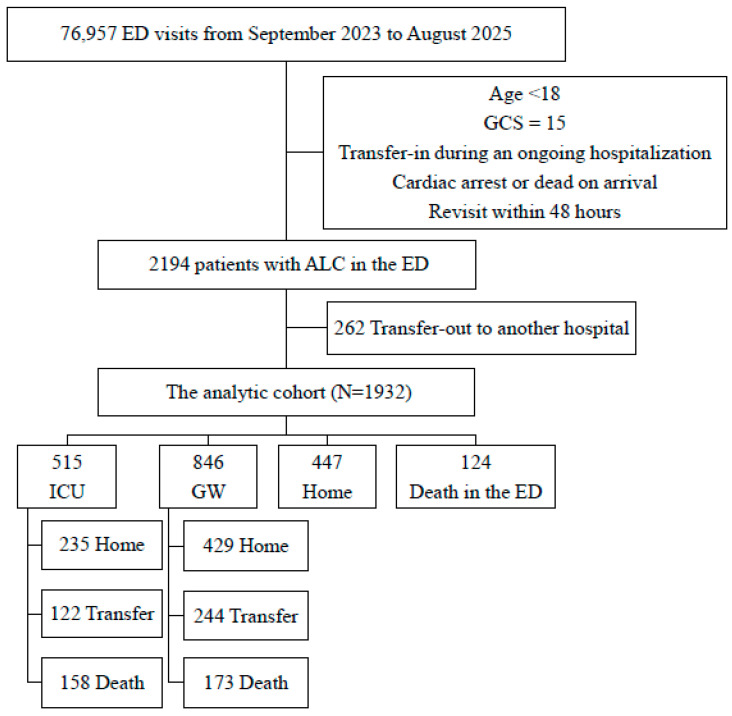
Flowchart of patients including dispositions and destinations. ED, emergency department; GCS, Glasgow Coma Scale; ALC, altered level of consciousness; ICU, intensive care unit; GW, general ward; Transfer, transferred to another hospital.

During the two-year study period (September 2023 to August 2025), 2194 ED visits met criteria for ALC (2.85% of 76,957 ED visits; Figure 1). After excluding 262 patients transferred to other hospitals from the ED, 1932 patients remained for outcome analyses. All variables required for the prespecified analyses were complete, and no participants were excluded due to missing data. In this analytic cohort, the mean age was 70.2 ± 15.7 years (median 74 years, interquartile range 61–82), and 902 patients (46.7%) were female. Older adults predominated, accounting for 60.1% of participants aged 70 years or older. The mean initial GCS score was 7.85 ± 3.24. Table 1 summarizes the ED course and early ED-course management intensity, including an ED length of stay of 17.6 h (interquartile range 7.1–27.4) and the use of vasopressors (289, 15.0%) or mechanical ventilation (401, 20.8%) within 1 h of ED arrival.

Transfer-out patients accounted for 262 of 2194 (11.9%) ALC visits. Compared with the analytic cohort (n = 1932), transfer-out patients were younger (64.4 ± 21.1 vs. 70.2 ± 15.7) and more often female (60.7% vs. 46.7%), had lower initial GCS scores (6.17 ± 3.22 vs. 7.85 ± 3.24), and had shorter ED length of stay (median 8.4 vs. 17.6 h). Early vasopressor use was similar between groups (14.1% vs. 15.0%), whereas mechanical ventilation within 1 h was substantially more frequent among transfer-out patients (47.7% vs. 20.8%). The etiologic distribution also differed markedly, with transfer-out cases enriched for toxic and undetermined etiologies (33.6% and 25.2%, respectively) and relatively fewer systemic infection and stroke cases (6.1% and 1.5%, respectively) (Supplementary Table S2).

**Figure 2:**
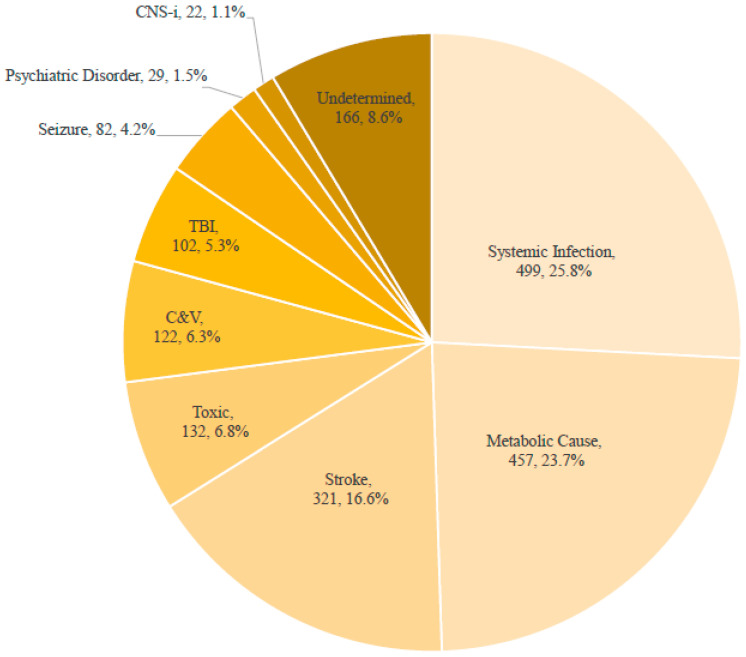
The etiologies of altered level of consciousness in the emergency department. C&V, cardiogenic and vascular cause; TBI, traumatic brain injury; CNS-i, central nervous system infection.Percentages may not total 100% due to rounding.

Detailed demographic and clinical characteristics are presented in Table 1. The clinical flow and ED disposition of the analytic cohort are summarized in Figure 1 and Supplementary Table S1, and the etiologic distribution of ALC is shown in Figure 2. Among the analytic cohort (N = 1932), 1361 patients (70.4%) were admitted to either a GW (846, 43.8%) or the ICU (515, 26.7%), 447 (23.1%) were discharged home from the ED, and 124 (6.4%) died in the ED. The most frequent etiologies were Systemic Infection (n = 499, 25.8%), Metabolic Cause (n = 457, 23.7%), and Stroke (n = 321, 16.6%). CNS-i (n = 22, 1.1%) and Psychiatric Disorder (n = 29, 1.5%) were relatively rare, while 166 cases (8.6%) were classified as Undetermined.

Overall mortality in the analytic cohort was 23.6% (455/1932), comprising ED mortality of 6.4% (124/1932) and in-hospital mortality of 24.3% (331/1361) among admitted patients (Table 2). Table 2 summarizes etiology-specific ED, in-hospital, and overall mortality with 95% CIs. Among all ED visits for ALC (N = 2194), 262 patients were transferred to other hospitals directly from the ED and therefore had unascertained outcomes. Under extreme-case assumptions, overall mortality ranged from 20.7% (455/2194; assuming 0% mortality among transfer-out patients) to 32.7% (717/2194; assuming 100% mortality). In the model-based sensitivity analysis (Supplementary Table S5), the mean predicted mortality risk among transfer-out patients was 20.7% (95% uncertainty interval [UI], 17.7–24.9), corresponding to 54.2 expected deaths (95% UI, 46.4–65.3) and an adjusted overall mortality of 23.2% (509.2/2194; 95% UI, 22.9–23.7) among all ALC ED visits. Mortality varied markedly by etiology, with the highest overall mortality observed for CNS-i (45.5%, 10/22), Stroke (32.1%, 103/321), and Systemic Infection (31.7%, 158/499), and the lowest for Toxic (6.8%, 9/132), Psychiatric Disorder (6.9%, 2/29), and Seizure (8.5%, 7/82). As expected, CIs were wider for less frequent etiologies, and these subgroup estimates should be interpreted cautiously.

Values are n/N (%; 95% CI). Overall mortality = ED death or in-hospital death (transfer-out excluded). In-hospital mortality was calculated among admitted patients only. 95% CIs were calculated using the Wilson score method.

In the multivariable logistic regression model for overall mortality (Table 3), etiology remained strongly associated with overall mortality (global *p* < 0.001 for etiologic categories). Compared with Systemic Infection, Metabolic Cause (adjusted OR 0.520, 95% CI 0.371–0.728), Toxic (0.239, 0.113–0.507), C&V (0.501, 0.296–0.849), TBI (0.336, 0.180–0.628), Seizure (0.262, 0.111–0.623), and Undetermined (0.580, 0.358–0.941) had lower adjusted odds of death, whereas Stroke did not differ significantly (1.085, 0.766–1.537). After accounting for etiologic category, older age (per year: 1.013, 95% CI 1.004–1.022), ICU admission (1.321, 1.001–1.744), vasopressor use (5.035, 3.747–6.766), and mechanical ventilation (1.506, 1.144–1.981) were associated with higher adjusted odds of death, whereas a higher initial GCS score was associated with lower adjusted odds (per point increase: 0.784, 0.751–0.819). Multicollinearity was low (all VIFs < 1.5). Model A showed moderate discrimination (AUC 0.749; bootstrap 95% CI 0.723–0.773; Supplementary Table S3), improved in Model B after adding vasopressor use and mechanical ventilation (AUC 0.795; bootstrap 95% CI 0.772–0.817; Table 3). Hosmer–Lemeshow *p*-values were 0.499 for Model A and 0.035 for Model B, with calibration plots shown in Supplementary Figure S1.

In the multivariable logistic regression model for ED mortality (Table 4), older age (per year: 1.028, 95% CI 1.013–1.044) and vasopressor use (18.428, 12.025–28.238) were associated with higher adjusted odds of ED death, whereas a higher initial GCS score was associated with lower adjusted odds (per point increase: 0.864, 0.805–0.929); mechanical ventilation was not statistically significant (1.420, 0.899–2.243). Model A showed limited discrimination for ED mortality (AUC 0.685; bootstrap 95% CI 0.637–0.733; Supplementary Table S4), whereas Model B showed markedly improved discrimination after adding vasopressor use and mechanical ventilation (AUC 0.869; bootstrap 95% CI 0.835–0.901; Table 4). Hosmer–Lemeshow *p*-values were 0.135 for Model A and 0.290 for Model B, with calibration plots shown in Supplementary Figure S2.

In prespecified supplementary analyses among admitted patients (GW + ICU), IPTW-ATT achieved good balance for age, sex, and initial GCS (all weighted |SMD| < 0.10; Supplementary Table S6). ICU admission was not associated with in-hospital mortality (HR 0.828, 95% CI 0.655–1.046; *p* = 0.113; N = 1348; Supplementary Table S7). Sensitivity analyses showed that key prognostic signals were robust to alternative GCS parameterizations: initial GCS remained strongly associated with in-hospital mortality when modeled continuously (HR 0.833, 95% CI 0.799–0.869; *p* < 0.001) or categorically (vs. GCS 3–6: GCS 7–10 HR 0.647, 95% CI 0.509–0.821; *p* < 0.001; GCS 11–14 HR 0.176, 95% CI 0.096–0.322; *p* < 0.001; Supplementary Table S8). Findings were also similar when the undetermined etiology category was included versus excluded (Supplementary Table S9). In interaction analyses using grouped etiologies, there was no evidence that the association between GCS and mortality differed by etiology group (all GCS × etiology interaction terms *p* > 0.20; Supplementary Table S10). Finally, competing-risks analyses using Fine–Gray models yielded consistent results, with older age, lower GCS, and vasopressor use remaining associated with worse in-hospital outcomes (Supplementary Table S11).

## 4. Discussion

In this retrospective cohort of adults presenting to a tertiary ED in South Korea, ALC accounted for 2.85% of all adult ED visits and was associated with substantial short-term mortality. Overall mortality was 23.6%, and notably, more than one-quarter of all deaths occurred in the ED (6.4% ED mortality; 27.3% of total deaths). Mortality in our cohort lies within, albeit toward the upper end of, the range reported in prior ED-based ALC and altered mental status studies (approximately 15–30%) [3,6,10]. Using the ALC-10 framework [9,10], systemic infection, metabolic causes, and stroke accounted for the majority of etiologies, and mortality differed markedly across categories. In multivariable analyses, early assessed clinical characteristics—particularly initial GCS score and vasopressor use—were strongly associated with both overall mortality and ED death.

The etiologic distribution observed here reinforces the syndromic nature of ALC and the clinical need for broad initial evaluation. The predominance of systemic infection and metabolic etiologies, together with the advanced age profile of the cohort, suggests that ALC in high-acuity ED settings is frequently driven by extracranial critical illness rather than isolated primary neurologic disorders, consistent with prior ED-based literature [3,4,5,6,7,8]. At the same time, stroke remained a common etiology. It carried high mortality, underscoring the importance of parallel pathways that ensure early stabilization while rapidly triaging for time-sensitive neurologic emergencies.

A significant contribution of this study is the explicit distinction between ED and in-hospital mortality. ED deaths comprised a meaningful fraction of total deaths, supporting the view that ED mortality is not merely an extension of in-hospital outcomes but may represent a distinct clinical trajectory characterized by extreme physiologic instability and limited time for diagnostic clarification. Prior population-based studies have shown that ED deaths are predominantly disease-related and often occur in the context of prolonged ED stays and end-of-life care needs, including frequent cardiopulmonary resuscitation [11]. Studies from other settings similarly report that ED death is not rare and tends to occur among patients requiring immediate high-acuity care [12,13]. Our findings extend this literature to ALC and indicate that ED mortality should be routinely reported and analyzed separately when evaluating ALC outcomes.

In IPTW-ATT-weighted Cox models balancing age, sex, and initial GCS, ICU (vs. GW) disposition was not associated with in-hospital mortality, suggesting that crude ICU–outcome associations primarily reflect differences in initial severity and case mix. These findings support interpreting ICU disposition as a marker of early clinical severity and case mix; this analysis does not provide a basis for attributing mortality differences to ICU disposition itself.

Etiology-specific patterns were also clinically informative. Compared with systemic infection, several categories showed substantially lower adjusted odds of death, including toxic and seizure-related ALC, which are often reversible with prompt recognition and supportive care. By contrast, stroke demonstrated mortality comparable to systemic infection after adjustment, suggesting that the overall risk burden in stroke-related ALC may remain high even when accounting for age, consciousness level, and early ED-course management-intensity markers. Despite its low frequency, CNS infection carried a very high overall mortality (45.5%), underscoring that delays in diagnostic work-up (e.g., neuroimaging and lumbar puncture when indicated) and initiation of antimicrobial therapy may have major consequences. These findings align with prior non-traumatic coma and ED death literature, in which infections, circulatory collapse, and acute neurologic catastrophes account for a large share of deaths. In contrast, isolated toxic exposures and primary psychiatric conditions rarely result in death when promptly recognized and treated [3,4,5,6,10,13].

Although we did not directly measure ED crowding or boarding, our results are compatible with the broader concern that system-level pressures may disproportionately affect outcomes in syndromic, high-acuity presentations such as ALC. Prior studies have linked ED crowding to short-term mortality and highlighted adverse outcomes among patients who remain in the ED while awaiting inpatient beds [14,15,16,17,18]. Future work should integrate operational metrics (e.g., crowding indices, time-to-ICU, and boarding duration) with ALC phenotyping to clarify how ED throughput constraints interact with physiologic severity and etiologic class to shape ED mortality and downstream outcomes.

The marked differences between transfer-out patients and the analytic cohort indicate that exclusion of transfer-out cases may introduce selection bias. Transfer-out patients had lower GCS scores and substantially higher early mechanical ventilation rates, suggesting greater initial illness severity. Yet, their etiologic profile was enriched. Accordingly, the net direction of selection bias is likely non-uniform. In this context, exclusion of transfer-out cases may have modestly overestimated overall mortality in the analyzed cohort, while the magnitude of associations between severity-related predictors and mortality may have been conservatively estimated.

This study has several strengths. We leveraged a large contemporary ED cohort, applied a structured, prespecified etiologic classification (ALC-10) adjudicated by a multidisciplinary consortium, and distinguished ED from in-hospital mortality to provide a more granular assessment of short-term outcomes. The multivariable models demonstrated low multicollinearity and good discrimination, with an AUC of 0.795 for overall mortality and 0.869 for ED mortality, supporting the internal coherence of the analyses.

Several limitations should be acknowledged. First, outcomes after direct inter-hospital transfer from the ED were not available, and transfer-out cases were excluded from the primary outcome analyses; this may introduce selection bias if disposition pathways differed systematically by severity or etiology. Treating transfer-out as a competing event would yield estimates for hospital mortality before transfer, but it cannot recover post-transfer deaths; therefore, we presented sensitivity analyses to characterize the potential impact of unobserved outcomes. To further assess the robustness of our findings, we performed a model-based sensitivity analysis. The mean predicted mortality risk among transfer-out patients was 20.7% (95% UI, 17.7–24.9), corresponding to 54.2 expected deaths (95% UI, 46.4–65.3) and an adjusted overall mortality estimate of 23.2% (95% UI, 22.9–23.7%). Nevertheless, these sensitivity analyses cannot substitute for direct outcome ascertainment after transfer, and residual selection bias related to disposition pathways may persist. Accordingly, early ED-course management-intensity markers should be interpreted as pragmatic proxies of early physiologic derangement and care intensity rather than as having direct causal effects on mortality. Second, IPTW analyses required trimming of extreme weights, which improves stability but may reduce generalizability to patients with limited overlap in initial risk profiles. Third, although Fine–Gray models addressed competing events, discharge alive and in-hospital transfer pathways may still introduce complexity that cannot be fully resolved without more granular time-to-intervention data and post-transfer outcomes. Fourth, although we used a definition of ALC and carefully screened all ED visits, our cohort necessarily excludes some patients with milder alterations in mental status who did not meet our criteria or whose symptoms were not documented as ALC in the medical record. The selection processes introduce potential biases that should be considered when interpreting the incidence and mortality estimates. Fifth, it is also important to recognize that delirium represents a multifactorial manifestation of acute brain dysfunction and altered mental status, often arising from the interaction between predisposing vulnerabilities and multiple precipitating insults. In this study, we classified patients based on medical records rather than syndromic constructs such as delirium. While this etiologic framework facilitates comparisons across ALC categories, it may oversimplify the complexity of delirium phenotypes in patients with overlapping causes, particularly in post-traumatic settings. The Undetermined category, by design, grouped together both truly unknown causes and mixed or overlapping etiologies not attributable to one primary category, which complicates the interpretation of its mortality profile. Our definition of CNS-i was deliberately conservative, requiring cerebrospinal fluid pleocytosis, and may therefore have underestimated early or atypical CNS-i with initially normal cerebrospinal fluid findings. Lastly, we did not have standardized information on baseline vulnerability, including comorbidity burden, premorbid functional status, pre-existing cognitive impairment or dementia, or code status (e.g., do-not-resuscitate orders). We also did not systematically measure ED crowding, boarding times, or specific time-to-treatment metrics, which precludes a more detailed analysis of system-level contributors to outcome.

In conclusion, ALC accounted for a significant proportion of adult ED visits and was associated with high mortality, with a substantial fraction of deaths occurring during ED care. Etiology and early severity markers—low initial GCS score and vasopressor requirement within 1 h of ED arrival—were strongly associated with both ED death and overall mortality. Because etiologic categories reflect the final ED diagnostic framework rather than a real-time triage label at presentation, etiologic comparisons should be interpreted as descriptive benchmarks across diagnostic groups. These findings support the clinical value of early risk stratification to prioritize diagnostic workup and to facilitate timely management and goals-of-care discussions.

## Figures and Tables

**Table 1:** Demographic characteristics of patients with altered level of consciousness.

New-Onset Altered Level of Consciousness in the Emergency Department	
Total ED visits	76,957
ALC cases	2194
Incidence of ALC (%)	2.85
**Excluding the Patients Transferred to Another Hospital (N = 1932)**	
Age (mean ± SD)	70.2 ± 15.7
GCS (mean ± SD)	7.85 ± 3.24
Female (n, %)	902 (46.7)
ED length of stay (hours) (median, IQR)	17.6 (7.1–27.4)
Vasopressor use within 1 h of ED arrival (n, %)	289 (15.0)
Mechanical ventilation within 1 h of ED arrival (n, %)	401 (20.8)
<40 years	107 (5.5)
40–49 years	95 (4.9)
50–59 years	214 (11.1)
60–69 years	355 (18.4)
70–79 years	525 (27.2)
80–89 years	559 (28.9)
≥90 years	77 (4.0)

Abbreviation: ED, emergency department; ALC, altered level of consciousness; SD, standard deviation; IQR, inter quartile range; GCS, Glasgow Coma Scale.

**Table 2:** Mortality by etiology (including ED and in-hospital deaths).

Etiology	ED Mortality	In-Hospital Mortality (Admitted)	Overall Mortality
Systemic Infection	48/499 (9.6%; 7.3–12.5)	110/366 (30.1%; 25.6–34.9)	158/499 (31.7%; 27.7–35.9)
Metabolic Cause	18/457 (3.9%; 2.5–6.1)	68/329 (20.7%; 16.6–25.4)	86/457 (18.8%; 15.5–22.7)
Stroke	18/321 (5.6%; 3.6–8.7)	85/275 (30.9%; 25.7–36.6)	103/321 (32.1%; 27.2–37.4)
Toxic	5/132 (3.8%; 1.6–8.6)	4/54 (7.4%; 2.9–17.6)	9/132 (6.8%; 3.6–12.5)
C&V	12/122 (9.8%; 5.7–16.4)	20/102 (19.6%; 13.1–28.4)	32/122 (26.2%; 19.2–34.7)
TBI	4/102 (3.9%; 1.5–9.7)	14/74 (18.9%; 11.6–29.3)	18/102 (17.6%; 11.5–26.2)
Seizure	0/82 (0.0%; 0.0–4.5)	7/59 (11.9%; 5.9–22.5)	7/82 (8.5%; 4.2–16.6)
Psychiatric Disorder	1/29 (3.4%; 0.6–17.2)	1/9 (11.1%; 2.0–43.5)	2/29 (6.9%; 1.9–22.0)
CNS-i	1/22 (4.5%; 0.8–21.8)	9/21 (42.9%; 24.5–63.5)	10/22 (45.5%; 26.9–65.3)
Undetermined	17/166 (10.2%; 6.5–15.8)	13/72 (18.1%; 10.9–28.5)	30/166 (18.1%; 13.0–24.6)
In total	124/1932 (6.4%; 5.4–7.6)	331/1361 (24.3%; 22.1–26.7)	455/1932 (23.6%; 21.7–25.5)

Abbreviation: ED, emergency department; C&V, cardiogenic and vascular cause; TBI, traumatic brain injury; CNS-i, central nervous systemi nfection.

**Table 3:** Multivariable logistic regression for overall mortality.

Variables	Adjusted OR	95% CI	*p*-Value
Etiology (Overall)	—	—	<0.001
Etiology, reference = Systemic Infection			
vs. Metabolic Cause	0.520	0.371–0.728	<0.001
vs. Stroke	1.085	0.766–1.537	0.647
vs. Toxic	0.239	0.113–0.507	<0.001
vs. C&V	0.501	0.296–0.849	0.010
vs. TBI	0.336	0.180–0.628	<0.001
vs. Seizure	0.262	0.111–0.623	0.002
vs. Psychiatric Disorder	0.505	0.113–2.257	0.371
vs. CNS-i	1.790	0.665–4.816	0.249
vs. Undetermined	0.580	0.358–0.941	0.027
Age (per 1 year)	1.013	1.004–1.022	0.004
GCS score (per 1 point)	0.784	0.751–0.819	<0.001
ICU admission, yes	1.321	1.001–1.744	0.049
Vasopressor use within 1 h of ED arrival, yes	5.035	3.747–6.766	<0.001
Mechanical ventilation use within 1 h of ED arrival, yes	1.506	1.144–1.981	0.003

Abbreviation: OR, odds ratio; CI, confidence interval; C&V, cardiogenic and vascular cause; TBI, traumatic brain injury; CNS-i, central nervous system infection; GCS, Glasgow Coma Scale; ICU, intensive care unit.

**Table 4:** Multivariable logistic regression for death in the emergency department.

Variables	Adjusted OR	95% CI	*p*-Value
Age (per 1 year)	1.028	1.013–1.044	<0.001
GCS score (per 1 point)	0.864	0.805–0.929	<0.001
Vasopressor use within 1 h of ED arrival, yes	18.428	12.025–28.238	<0.001
Mechanical ventilation use within 1 h of ED arrival, yes	1.420	0.899–2.243	0.133

Abbreviation: OR, odds ratio; CI, confidence interval; GCS, Glasgow Coma Scale.

## Data Availability

The de-identified individual-level dataset and data dictionary are provided as Supplementary Materials.

## Supplementary Materials

The following supporting information can be downloaded at: https://www.mdpi.com/article/10.3390/jcm15052037/s1, Table S1. Disposition and post-admission outcomes by etiology; Table S2. ED-presentation characteristics of transfer-out patients compared with the analytic cohort; Table S3. Multivariable logistic regression model (Model A) using ED-arrival and ED-disposition markers for overall mortality; Table S4. Multivariable logistic regression model (Model A) using ED-arrival markers for ED mortality; Table S5. Model-based sensitivity analysis for transfer-out patients: expected deaths and adjusted overall mortality; Table S6. Covariate balance before and after IPTW-ATT weighting among admitted patients (ICU vs. GW); Table S7. IPTW (ATT)-weighted Cox proportional hazards model for in-hospital mortality (ICU vs. GW); Table S8. Sensitivity analysis: alternative parameterization of initial GCS in IPTW (ATT)-weighted Cox models; Table S9. Sensitivity analysis: handling of the Undetermined etiology category in Cox models for in-hospital mortality; Table S10. Interaction analysis: GCS × etiology (grouped) in Cox proportional hazards models for in-hospital mortality (admitted patients); Table S11. Competing-risks analysis (Fine–Gray) for in-hospital mortality with discharge alive and in-hospital transfer as competing events; Figure S1. Calibration plot for overall mortality. Abbreviations: ED, emergency department; GCS, Glasgow Coma Scale; ICU, intensive care unit. The dashed diagonal line denotes perfect calibration. Model A (ED-arrival and ED-disposition markers) is shown with a solid line; Model B (Model A covariates plus early ED-course management-intensity markers) is shown with a dotted line. Figure S2. Calibration plot for ED mortality prediction models (Model A vs. Model B). Abbreviations: ED, emergency department; GCS, Glasgow Coma Scale. Calibration plot (ED mortality). Model A included ED-arrival markers (age and initial GCS), and Model B additionally included early ED-course management-intensity markers (vasopressor use and mechanical ventilation within 1 h of ED arrival). Calibration is shown across deciles of predicted risk.

## Abbreviations

The following abbreviations are used in this manuscript:

ALC	Altered Level of Consciousness
ALC-10	a ten-category etiologic framework to characterize the distribution of altered level of consciousness
ATT	average treatment effect on the treated
CNS-i	Central Nervous System Infection
CI	Confidence Interval
COVID-19	coronavirus disease-2019
C&V	Cardiogenic and Vascular cause
ED	Emergency Department
GCS	Glasgow Coma Scale
GW	General Ward
ICU	Intensive Care Unit
IPTW	inverse probability of treatment weighting
OR	Odds Ratio
TBI	Traumatic Brain Injury
UI	uncertainty interval
