# Supplementary material for: Altered Level of Consciousness in a Tertiary Emergency Department: Etiologies, Mortality, and Outcomes
Source: J Clin Med. 2026 Mar 7;15(5):2037. doi: 10.3390/jcm15052037 (PMC12985886; doi:10.3390/jcm15052037)
Supplement: Supplementary file 1 [file jcm-15-02037-s001.zip › jcm-4073664-supplementary.pdf]

Supplementary Table S1. Disposition and post-admission outcomes by etiology

Etiology	N (%)	GW, n (%)	Discharge from GW	ICU, n (%)	Discharge from ICU	Home, n (%)	Death in ED, n (%)
Systemic Infection	499 (25.8)	269 (53.9)	Transfer 90<br>Home 105<br>Death 74	97 (19.4)	Transfer 31<br>Home 30<br>Death 36	85 (17.0)	48 (9.6)
Metabolic Cause	457 (23.7)	259 (56.7)	Transfer 60<br>Home 149<br>Death 50	70 (15.3)	Transfer 13<br>Home 39<br>Death 18	110 (24.1)	18 (3.9)
Stroke	321 (16.6)	100 (31.2)	Transfer 38<br>Home 36<br>Death 26	175 (54.5)	Transfer 29<br>Home 87<br>Death 59	28 (8.7)	18 (5.6)
Toxic	132 (6.8)	38 (28.8)	Transfer 10<br>Home 27<br>Death 1	16 (12.1)	Transfer 8<br>Home 5<br>Death 3	73 (55.3)	5 (3.8)
C&V	122 (6.3)	41 (33.6)	Transfer 16<br>Home 19<br>Death 6	61 (50.0)	Transfer 16<br>Home 31<br>Death 14	8 (6.6)	12 (9.8)
TBI	102 (5.3)	20 (19.6)	Transfer 3<br>Home 16<br>Death 1	54 (52.9)	Transfer 11<br>Home 30<br>Death 13	24 (23.5)	4 (3.9)
Seizure	82 (4.2)	49 (59.8)	Transfer 11<br>Home 35<br>Death 3	10 (12.2)	Transfer 3<br>Home 3<br>Death 4	23 (28.0)	0 (0.0)
Psychiatric Disorder	29 (1.5)	9 (31.0)	Transfer 3<br>Home 5<br>Death 1	0 (0.0)	Transfer 0<br>Home 0<br>Death 0	19 (65.5)	1 (3.4)
CNS-i	22 (1.1)	9 (40.9)	Transfer 1<br>Home 6<br>Death 2	12 (54.5)	Transfer 4<br>Home 1<br>Death 7	0 (0.0)	1 (4.5)
Undetermined	166 (8.6)	52 (31.3)	Transfer 12<br>Home 31<br>Death 9	20 (12.0)	Transfer 7<br>Home 9<br>Death 4	77 (46.4)	17 (10.2)

Abbreviations: GW, general ward; ICU, intensive care unit; ED, emergency department; C&V, cardiogenic and vascular cause; TBI, traumatic brain injury; CNS-i, central nervous system infection; Transfer: transferred to another hospital.

Percentages in GW/ICU/Home/Death in ED columns are row percentages using N of each etiology as the denominator.

Supplementary Table S2. ED-presentation characteristics of transfer-out patients compared with the analytic cohort

Variable	Analytic cohort (n=1,932)	Transfer-out (n=262)	P value
Age, years, mean ± SD	70.2 ± 15.7	64.4 ± 21.1	<0.001
Age, years, median (IQR)	74 (61–82)	68.5 (50–83)	N/A
Female, n (%)	902 (46.7%)	159 (60.7%)	<0.001
Initial GCS score, mean ± SD	7.85 ± 3.24	6.17 ± 3.22	<0.001
Initial GCS score, median (IQR)	8 (5–10)	6 (3–8)	N/A
ED length of stay, h, median (IQR)	17.6 (7.1–27.4)	8.4 (5.0–14.7)	<0.001
Vasopressor use within 1 h, n (%)	289 (15.0%)	37 (14.1%)	0.791
Mechanical ventilation within 1 h, n (%)	401 (20.8%)	125 (47.7%)	<0.001
Etiology distribution, n (%)			Global p <0.001
Systemic infection	499 (25.8%)	16 (6.1%)	
Metabolic cause	457 (23.7%)	48 (18.3%)	
Stroke	321 (16.6%)	4 (1.5%)	
C&V	122 (6.3%)	9 (3.4%)	
Seizure	82 (4.2%)	15 (5.7%)	
Toxic	132 (6.8%)	88 (33.6%)	
Psychiatric disorder	29 (1.5%)	10 (3.8%)	
CNS-i	22 (1.1%)	1 (0.4%)	
TBI	102 (5.3%)	5 (1.9%)	
Undetermined	166 (8.6%)	66 (25.2%)	

Abbreviations: ED, emergency department; SD, standard deviation; IQR, interquartile range; GCS, Glasgow Coma Scale; C&V, cardiogenic and vascular cause; CNS-i, central nervous system infection; TBI, traumatic brain injury.

The global p value for etiology distribution was obtained from a chi-square test across the 10 categories.

Supplementary Table S3. Multivariable logistic regression model (Model A) using ED-arrival and ED-disposition markers for overall mortality

Variable	Adjusted OR (95% CI)	p-value
Etiology (Overall)	—	<0.001
Etiology, reference = Systemic Infection		
vs. Metabolic cause	0.551 (0.400–0.760)	<0.001
vs. Stroke	1.157 (0.826–1.620)	0.396
vs. C&V	0.755 (0.467–1.221)	0.252
vs. Seizure	0.257 (0.112–0.590)	0.001
vs. Toxic	0.249 (0.120–0.517)	<0.001
vs. Psychiatric disorder	0.659 (0.151–2.877)	0.582
vs. CNS-i	1.665 (0.628–4.417)	0.310
vs. TBI	0.466 (0.261–0.833)	0.010
vs. Undetermined	0.777 (0.491–1.231)	0.284
Age (per 1 year)	1.024 (1.015–1.034)	<0.001
Initial GCS score (per 1 point)	0.779 (0.747–0.812)	<0.001
ICU admission, yes	1.906 (1.495–2.431)	<0.001

Abbreviations: OR, odds ratio; CI, confidence interval; ED, emergency department; GCS, Glasgow Coma Scale; ICU, intensive care unit; C&V, cardiogenic and vascular cause; CNS-i, central nervous system infection; TBI, traumatic brain injury.

Supplementary Table S4. Multivariable logistic regression model (Model A) using ED-arrival markers for ED mortality

Variable	Adjusted OR (95% CI)	p-value
Age (per 1 year)	1.019 (1.005–1.034)	0.007
Initial GCS score (per 1 point)	0.815 (0.763–0.872)	<0.001

Abbreviations: ED, emergency department; OR, odds ratio; CI, confidence interval; GCS, Glasgow Coma Scale.

Supplementary Table S5. Model-based sensitivity analysis for transfer-out patients: expected deaths and adjusted overall mortality

Metric	Value
Analytic cohort, N	1,932
Observed deaths in analytic cohort, n	455
Transfer-out patients (inter-hospital transfer from the ED), N	262
Mean predicted mortality risk among transfer-out patients, %	20.7 (17.7 – 24.9)
Expected deaths among transfer-out patients, n	54.2 (46.4 – 65.3)
Total deaths (observed + expected), n	509.2 (501.4 – 520.3)
Adjusted overall mortality among all ED visits for ALC (N=2,194), %	23.2 (22.9 – 23.7)

Abbreviations: ALC, altered level of consciousness; ED, emergency department; GCS, Glasgow Coma Scale.

Overall mortality was defined as death in the ED or during the hospitalization of the patients with observed outcomes. The model-based sensitivity analysis used a multivariable logistic regression model fitted in patients with observed outcomes and applied to transfer-out patients to estimate expected deaths. Values in parentheses represent 95% uncertainty intervals (UI) from 10,000 simulations; UIs correspond to the 2.5 – 97.5 percentiles. The expected number of deaths among transfer-out patients was calculated as the sum of predicted probabilities, and the adjusted overall mortality among all ED visits for ALC (N=2,194) was calculated as: $\frac{(\text{Observed deaths in analytic cohort} + \text{Expected deaths in transfer-out})}{2{,}194}$

Supplementary Table S6. Covariate balance before and after IPTW-ATT weighting among admitted patients (ICU vs. GW)

Covariate	ICU (unweighted, n-512)	GW (unweighted, n=836)	SMD (unweighted)	ICU (weighted, IPTW-ATT)	GW (weighted, IPTW-ATT)	SMD (weighted)
Age (years), mean±SD	68.04±14.93	72.23±14.19	-0.287	68.04±14.91	67.52±16.63	0.033
Sex (male)	287 (56.1%)	437 (52.3%)	0.076	56.1%	56.4%	-0.008
Initial GCS score	7.09±2.97	7.74±3.14	-0.210	7.09±2.97	7.06±3.04	0.012

Abbreviations: IPTW, inverse probability of treatment weighting; ATT, average treatment effect in the treated; SMD, standardized mean difference; ICU, inten sive care unit; GW, general ward; SMD, standardized mean differences; SD, standard deviation; GCS, Glasgow Coma Scale.

Balance diagnostics for the propensity-score (PS)–weighted admitted cohort (GW+ICU). The treatment group was ICU admission and the comparison group was GW admission. The PS model included age (years), sex (male), and initial GCS score at emergency department presentation. IPTW targeting the ATT was applied; extreme weights were trimmed according to the prespecified rule. Continuous variables are shown as mean ± SD, and categorical variables as n (%). SMDs are presented for unweighted and weighted samples; absolute SMD < 0.10 indicates adequate covariate balance.

Supplementary Table S7. IPTW (ATT)-weighted Cox proportional hazards model for in-hospital mortality (ICU vs. GW)

Model	Predictor	N	Events	HR	95% CI	p-value
IPTW(ATT)-weighted Cox	ICU admission (vs. GW admission)	1348	331	0.828	0.655 – 1.046	0.113

Abbreviations: IPTW, inverse probability of treatment weighting; ATT, average treatment effect in the treated; ICU, intensive care unit; GW, general ward; HR, hazard ratio; CI, confidence interval; GCS, Glasgow Coma Scale.

IPTW(ATT)-weighted Cox proportional hazards regression for in-hospital mortality among admitted patients (GW+ICU). Time scale was days of hospitalization. The event was death after hospitalization; discharge alive and in-hospital transfer were treated as censoring events. ICU admission (vs. GW admission) was the exposure of interest. IPTW(ATT) weights were derived from the PS model (age, sex, and initial GCS at emergency department presentation), with pre-specified trimming of extreme weights. HRs with 95% CIs and two-sided p-values are reported; robust (sandwich) standard errors were used.

Supplementary Table S8. Sensitivity analysis: alternative parameterization of initial GCS in IPTW (ATT)-weighted Cox models

Model	Predictor	N	Events	HR	95% CI	p-value
Weighted Cox (GCS continuous)	ICU (vs. GW)	1348	331	0.904	0.715 – 1.114	0.403
	Age (per 1-year increase)	1348	331	1.012	1.004 – 1.020	0.004
	Sex (male) (vs. female)	1348	331	1.199	0.954 – 1.507	0.120
	Initial GCS (per 1-point increase)	1348	331	0.833	0.799 – 0.869	<0.001
Weighted Cox (GCS categorial)	ICU (vs. GW)	1348	331	0.890	0.703 – 1.126	0.332
	Age (per 1-year increase)	1348	331	1.012	1.004 – 1.020	0.005
	Sex (male) (vs. female)	1348	331	1.210	0.962 – 1.522	0.103
	Initial GCS	1348	331	Reference		
	Initial GCS 7 – 10 (vs. 3-6)	1348	331	0.647	0.509 – 0.821	<0.001
	Initial GCS 11 – 14 (vs. 3-6)	1348	331	0.176	0.096 – 0.322	<0.001

Abbreviations: GCS, Glasgow Coma Scale; HR, hazard ratio; CI, confidence interval; IPTW, inverse probability of treatment weighting; ATT, average treatme nt effect in the treated; ICU, intensive care unit; GW, general ward.

Sensitivity analysis for the IPTW (ATT)-weighted Cox model (admitted cohort, GW+ICU) evaluating alternative parameterizations of initial GCS. Time scale was days of hospitalization; the event was death after admission; discharge alive and in-hospital transfer were treated as censoring events. Results are shown for (1) GCS modeled as a continuous variable and (2) GCS modeled categorically (3–6, 7–10, 11–14), with 3–6 as the reference category. IPTW (ATT) wei ghts were derived from the PS model (age, sex, and initial GCS), with prespecified trimming of extreme weights. HRs with 95% CIs and two-sided p-value s are reported; robust (sandwich) standard errors were used.

Supplementary Table S9. Sensitivity analysis: handling of the Undetermined etiology category in Cox models for in-hospital mortality

Model	Predictor	N	Events	HR	95% CI	p-value
Cox (10-category of etiology; includes Undetermined)	Age (per 1-year increase)	1361	331	1.012	1.004 – 1.020	0.005
	Initial GCS (per 1-point increase)	1361	331	0.829	0.796 – 0.863	<0.001
	Etiology: Metabolic Cause (vs. Systemic Infection)	1361	331	1.012	0.741 – 1.384	0.939
	Etiology: Stroke (vs. Systemic Infection)	1361	331	1.057	0.780 – 1.434	0.720
	Etiology: C&V (vs. Systemic Infection)	1361	331	0.722	0.437 – 1.193	0.204
	Etiology: Seizure (vs. Systemic Infection)	1361	331	1.036	0.565 – 1.900	0.909
	Etiology: Toxic (vs. Systemic Infection)	1361	331	0.433	0.183 – 1.027	0.057
	Etiology: Psyhiatric Disorder (vs. Systemic Infection)	1361	331	1.234	0.187 – 8.127	0.827
	Etiology: CNS infection (vs. Systemic Infection)	1361	331	0.703	0.374 – 1.320	0.273
	Etiology: TBI (vs. Systemic Infection)	1361	331	0.446	0.252 – 0.786	0.005
	Etiology: Undetermined (vs. Systemic Infection)	1361	331	0.601	0.340 – 1.062	0.080
	ICU admission (vs. GW)	1361	331	0.893	0.699 – 1.141	0.366
	Vasopressor use	1361	331	1.478	1.104 – 1.978	0.009
	Mechanical ventilation	1361	331	1.213	0.955 – 1.541	0.113
Cox (9-category of etiology; excludes Undetermined)	Age (per 1-year increase)	1289	318	1.012	1.004 – 1.021	0.006
	Initial GCS (per 1-point increase)	1289	318	0.831	0.797 – 0.866	<0.001
	Etiology: Metabolic Cause (vs. Systemic Infection)	1289	318	1.017	0.744 – 1.391	0.917
	Etiology: Stroke (vs. Systemic Infection)	1289	318	1.049	0.772 – 1.424	0.761
	Etiology: C&V (vs. Systemic Infection)	1289	318	0.719	0.435 – 1.190	0.199
	Etiology: Seizure (vs. Systemic Infection)	1289	318	1.050	0.573 – 1.924	0.876
	Etiology: Toxic (vs. Systemic Infection)	1289	318	0.432	0.181 – 1.031	0.059
	Etiology: Psychiatric Disorder (vs. Systemic Infection)	1289	318	1.241	0.188 – 8.203	0.822
	Etiology: CNS infection (vs. Systemic Infection)	1289	318	0.702	0.371 – 1.330	0.281
	Etiology: TBI (vs. Systemic Infection)	1289	318	0.451	0.254 – 0.802	0.007
	ICU admission (vs. GW)	1289	318	0.905	0.707 – 1.159	0.435
	Vasopressor use	1289	318	1.489	1.109 – 1.999	0.008

Mechanical ventilation	1289	318	1.231	0.962 – 1.575	0.099

Abbreviations: ALC, altered level of consciousness; HR, hazard ratio; CI, confidence interval; ICU, intensive care unit; GW, general ward; ED, emergency d epartment; GCS, Glasgow Coma Scale.

Sensitivity analysis for etiologic adjustment in time-to-event models among admitted patients (GW+ICU). Cox proportional hazards models were fitted for in-hospital mortality using days of hospitalization as the time scale. The event was death after admission; discharge alive and in-hospital transfer were treated a s censoring events. Etiology was modeled using the ALC-10 framework as indicator variables with systemic infection as the reference group. Two specificati ons are shown: (1) including the Undetermined category and (2) excluding Undetermined cases from the analysis. HRs with 95% CIs and two-sided p-values are reported.

Supplementary Table S10. Interaction analysis: GCS × etiology (grouped) in Cox proportional hazards models for in-hospital mortality (admitted patients)

Model	Predictor	N	Events	HR	95% CI	p-value
GLOBAL TEST	P for interaction	1361	331			0.508
Cox with GCS × Etiology interaction (grouped; robust standard error)	Age (per 1-year increase)	1361	331	1.013	1.004 – 1.021	0.003
	Sex (male) (vs. female)	1361	331	1.206	0.961 – 1.515	0.107
	Initial GCS (per 1-point increase)	1361	331	0.843	0.784 – 0.907	<0.001
	Etiology: Metabolic Cause (vs. Systemic Infection)	1361	331	0.873	0.408 – 1.868	0.726
	Etiology: Stroke (vs. Systemic Infection)	1361	331	1.291	0.618 – 2.701	0.497
	Etiology, Others (vs. Systemic Infection)	1361	331	0.908	0.454 – 1.815	0.784
	ICU admission (vs. GW)	1361	331	0.874	0.685 – 1.115	0.279
	Vasopressor use	1361	331	1.476	1.097 – 1.987	0.010
	Mechanical ventilation	1361	331	1.218	0.959 – 1.547	0.106
	Interaction: GCS × Metabolic Cause	1361	331	1.027	0.917 – 1.149	0.648
	Interaction: GCS × Stroke	1361	331	0.974	0.877 – 1.082	0.626
	Interaction: GCS × Other	1361	331	0.933	0.832 – 1.046	0.232

Abbreviations: GCS, Glasgow Coma Scale; HR, hazard ratio; CI, confidence interval; ICU, intensive care unit; GW, general ward.

Interaction analysis was performed using a Cox proportional hazards model with days of hospitalization as the time scale; death after admission was the eve nt, and discharge alive or in-hospital transfer was treated as censoring. Etiology was grouped as Infection (reference), Metabolic, Stroke, and Other to stabili ze estimation. The interaction model included interaction terms between initial GCS and etiology group (GCS×Metabolic, GCS×Stroke, and GCS×Other). "P f or interaction" denotes the global p-value for the set of interaction terms, obtained from a likelihood ratio test comparing the reduced model (without interac tion terms) and the interaction model including the interaction term (df = 3). Hazard ratios (HRs) with 95% confidence intervals (CIs) are reported with rob ust (sandwich) standard errors.

Supplementary Table S11. Competing-risks analysis (Fine–Gray) for in-hospital mortality with discharge alive and in-hospital transfer as competing events

Model	Variable	N	Events	sHR	95% CI	p-value
Fine–Gray sub-distribution hazards	Age (per 1-year increase)	1361	331	1.011	1.003 – 1.019	0.007
	Initial GCS (per 1-point increase)	1361	331	0.831	0.799 – 0.865	<0.001
	Etiology: Metabolic Cause (vs. Systemic Infection)	1361	331	1.005	0.739 – 1.368	0.973
	Etiology: Stroke (vs. Systemic Infection)	1361	331	1.061	0.785 – 1.433	0.702
	Etiology, Others (vs. Systemic Infection)	1361	331	0.619	0.456 – 0.840	0.002
	ICU admission (vs. GW)	1361	331	0.875	0.687 – 1.113	0.276
	Vasopressor use	1361	331	1.469	1.103 – 1.956	0.009
	Mechanical ventilation	1361	331	1.232	0.973 – 1.560	0.084

Abbreviations: sHR, subdistribution hazard ratio; CI, confidence interval; ICU, intensive care unit; GW, general ward.

Competing-risks regression for in-hospital mortality among admitted patients (GW+ICU) using the Fine–Gray subdistribution hazards model. Time scale was d ays of hospitalization. The event of interest was death after admission; discharge alive and in-hospital transfer were treated as competing events. Subdistributi on hazard ratios (sHRs) with 95% CIs and two-sided p-values are reported. Etiology was modeled as grouped categories (Systemic Infection as the reference group).

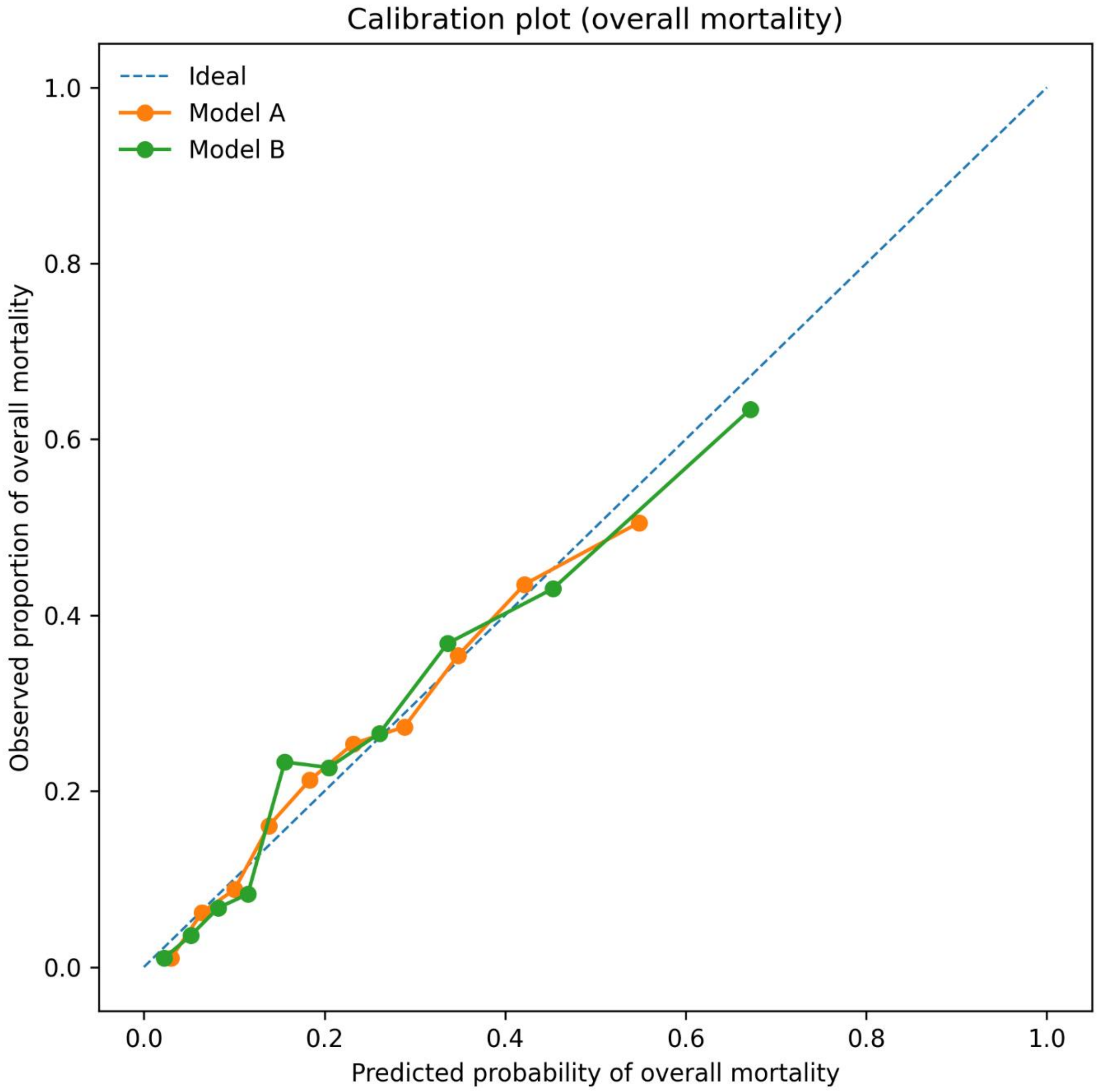

Supplementary Figure S1. Calibration plot for overall mortality

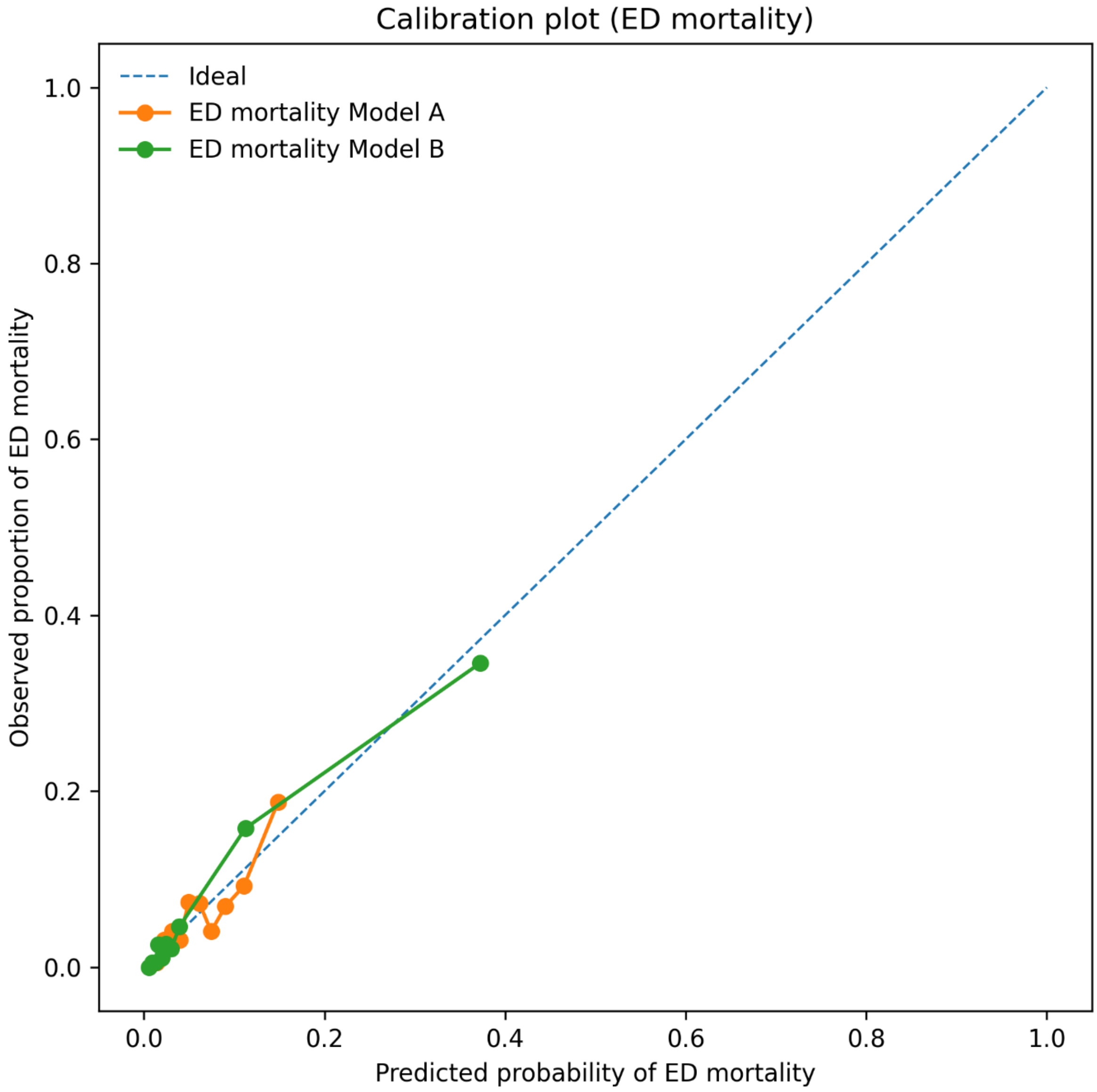

Supplementary Figure S2. Calibration plot for ED mortality prediction models (Model A vs. Model B)
